# A Machine-Learning-Based Approach for Railway Track Monitoring Using Acceleration Measured on an In-Service Train

**DOI:** 10.3390/s23177568

**Published:** 2023-08-31

**Authors:** Abdollah Malekjafarian, Chalres-Antoine Sarrabezolles, Muhammad Arslan Khan, Fatemeh Golpayegani

**Affiliations:** 1 Structural Dynamics and Assessment Laboratory, School of Civil Engineering, University College Dublin, D04V1W8 Dublin, Ireland; 2 The École Nationale des Travaux Publics de l’État (ENTPE), 69518 Lyon, France; 3 School of Computer Science, University College Dublin, D04V1W8 Dublin, Ireland

**Keywords:** machine learning, railway infrastructure monitoring, track damage detection, SHM, acceleration, in-service train measurements, drive-by monitoring, ANN

## Abstract

In this paper, a novel railway track monitoring approach is proposed that employs acceleration responses measured on an in-service train to detect the loss of stiffness in the track sub-layers. An Artificial Neural Network (ANN) algorithm is developed that works with the energies of the train acceleration responses. A numerical model of a half-car train coupled with a track profile is employed to simulate the train vertical acceleration. The energy of acceleration signals measured from 100 traversing trains is used to train the ANN for healthy track conditions. The energy is calculated every 15 m along the track, each of which is called a slice. In the monitoring phase, the trained ANN is used to predict the energies of a set of train crossings. The predicted energies are compared with the simulated ones and represented as the prediction error. The damage is modeled by reducing the soil stiffness at the sub-ballast layer that represents hanging sleepers. A damage indicator (DI) based on the prediction error is proposed to visualize the differences in the predicted energies for different damage cases. In addition, a sensitivity analysis is performed where the impact of signal noise, slice sizes, and the presence of multiple damaged locations on the performance of the DI is assessed.

## 1. Introduction

In recent years, with the increasing global demand for mass transportation and freight, the maintenance of existing transport infrastructure has become important. Railway systems are vital components of transportation systems, which provide a reliable, cost-efficient, and sustainable transportation mode. Railway services are commonly seen as a safe mode of travel (or freight moving) with low tariffs, reliable speeding, and low environmental impact [1]. Most of the existing railway infrastructure is aged and requires continuous monitoring to keep it in service, which requires enormous cost [2]. Moreover, these structures are subjected to heavier axle loads, faster train speeds, and greater frequencies of trains, which have resulted in rapid deterioration over time [3]. Hence, efficient and reliable infrastructure monitoring systems are needed to ensure these systems run smoothly at a reasonable cost.

Railways tracks are the first contact between the heavy, traversing trains and the railway structure below. For that reason, the track receives significant amounts of stresses (both longitudinal and transverse), resulting in severe deterioration over time [3,4,5,6,7,8]. The structural condition of the railway tracks needs to be monitored regularly and essential repairs need to be planned to provide an effective first train–rail contact. Conventionally, railway tracks are monitored through regular visual inspection by walking along the track with the help of hand-held surveying equipment, e.g., tachometer or leveler. This method is expensive to perform frequently and intensive when the whole network is considered [9,10]. Moreover, this method detects only major faults that are visually detectable, and it is not efficient on detecting inner damages, which are only apparent when a train passes over. Another commonly implemented method for monitoring tracks is the use of a specialized vehicle, i.e., Track Recording Vehicle (TRV), which is equipped with optical and inertial sensors to collect track geometric information while traversing [11]. These vehicles can measure the level and alignment of the rail, track gauge, cross level, rail twist, curvature/curve radius, gradient, and track position, using GPS and displacement sensors [11,12]. According to the European regulations, the geometric characterization of the railway track has to be measured at a regular frequency in accordance with the EN13848 standard [12], which can be implemented using TRVs. If any characteristic is above the tolerance limit, temporary speed restrictions or a suspension of train operations may be applied. Although TRVs provide reliable inspection data, they are extremely expensive to deploy, and they create traffic disruptions during inspection in the form of occupied tracks and restricted traffic speeds.

Recent research on the use of sensing technology and computational power has opened new opportunities to improve railway track monitoring systems and to provide accurate and cost-effective solutions [9,13]. A common practice is the installation of sensors on the track and monitoring track accelerations to detect any faults. For example, Liu et al. [14] proposed a method that uses a distributed network of sensors installed on the track, which measures vertical accelerations and strains of the track. Similarly, many researchers have proposed the use of fiber Bragg grating (FBG) strain sensors to monitor the behavior of the track [15,16]. Although direct track monitoring systems yield effective results, these are not feasible for the complex and widely spread network of railway infrastructure [3].

Indirect methods of monitoring have received much attention in recent years, which are based on the installation of sensors on a passing train for track health monitoring and railway bridge monitoring, also known as “drive-by” monitoring [17,18,19,20,21]. In these methods, train components closer to the track, e.g., suspensions, are instrumented to measure their dynamic responses, which can also be real-time data of a passing train. In this way, several passenger trains can be instrumented and used for continuous railway track monitoring, which can detect damage at its early stage. Several types of sensors, like laser technology [22], cameras [23,24], and inertial sensors [25], have been tested to develop drive-by track monitoring systems. Lederman et al. [20] proposed an energy-based method to inspect track changes using acceleration from the train body. They used the energy of the signals measured from several passes of an in-service train and employed a feature detection indicator to identify changes in the track over time. In order to find the track stiffness profile, Nafari et al. [22] used the relative vertical distance between the rail surface at two points measured from the train, which can be acquired using a laser-based rolling deflection measurement system. Although these approaches have shown efficacy in the results, they have targeted one specific damage case for analysis. Malekjafarian et al. [26] employed the Hilbert–Huang transformation in order to obtain the instantaneous amplitudes of the acceleration signals measured on a train. They proposed a representation of the energy of the signal as a function of train localization to detect track irregularities. Malekjafarian et al. [3] showed that the bogie-filtered displacement (BFD) can be numerically obtained using the measured drive-by acceleration and the system was validated using an in-service Irish rail train. It is also seen that the BFD is sensitive to train speed and signal noise [3]. However, with the use of statistics of train passes with different forward speeds, BFD can effectively detect the loss of stiffness on-track.

Several approaches have been made to model the train–track interaction [27,28]. OBrien et al. [28] proposed a method for inferring a track longitudinal profile from drive-by measurement using a numerical model. The train was modeled as a 10-degree-of-freedom (DOF) half-train and the track was modeled as a beam resting on a three-layer sprung mass system representing the ballast, pads, and sleepers. OBrien et al. [25] compared the results between the numerical simulations and field test measurements to validate the modeling scheme. They used an uneven longitudinal track profile for realistic representation of the track–train interaction. Using this method, dynamic responses of the train as well as railway structures can be measured with the help of numerical methods [29].

Machine learning techniques have recently been shown to be a feasible approach for drive-by monitoring of railway tracks. These algorithms create a neural network model that needs to be trained using the known data and parameters in time from a benchmark condition of a structure. Then, the model is tested using the data with unknown condition information with the help of hidden layers and neurons [30,31]. There have been significant developments in the application of machine learning techniques to SHM and damage detection. Avci et al. [30] described a comprehensive overview of the use of machine learning for vibration-based damage detection methods that can be implemented for any type of structure. Bridge structures have commonly been studied for developing SHM systems using machine learning algorithms [32]. Neves et al. [33] and Gonzalez and Karoumi [34] applied an Artificial Neural Network (ANN) that was trained using railway bridge accelerations to identify structure behavior and to develop damage detection systems using long-term data. Santos et al. [35] used a machine learning algorithm on bridge inspection data and identified inaccuracies caused by inspection issues. The effect of temperature changes on bridges was also studied using the Kalman-filter-based ANN, which has shown to eliminate the temperature effects for bridge health monitoring [36]. There are many researchers who have used cluster-based and data-driven approaches for bridge health monitoring [37,38,39,40] and have shown promising results for effective monitoring systems. Malekjafarian et al. [31,41] recently applied the concepts of machine learning on drive-by monitoring of the structures. They proposed the use of an ANN model using vehicle data, which can detect bridge frequencies and cracks on the deck. However, the road profile roughness and temperature effects have shown sensitivity to the drive-by approaches. Similarly, the learning-based approaches have been implemented for railway track monitoring, e.g., to detect and classify the severity of rail corrugation [42], wheelflat [43], and track profile [6,25,26,44], and have produced reasonable results for detecting track damages.

In this paper, a novel two-stage railway track condition monitoring approach is developed using the responses measured on a passing train and a machine learning algorithm. In the first stage, an ANN is created and trained using the energy of the accelerations collected from a fleet of trains passing over a healthy track at different forward speeds. The trained ANN is then used to predict the energy of the signals in the monitoring phase. The prediction error, which is the difference between the predicted and the simulated accelerations from each train pass, is used as the damage indicator. In the second stage, a Gaussian fitting method is applied to the prediction errors under healthy and damaged conditions. This process improves the damage detection capabilities of the system and has been shown to effectively detect damaged track conditions by reasonably increasing the fitted prediction error.

## 2. Numerical Modeling

**Figure 1:**
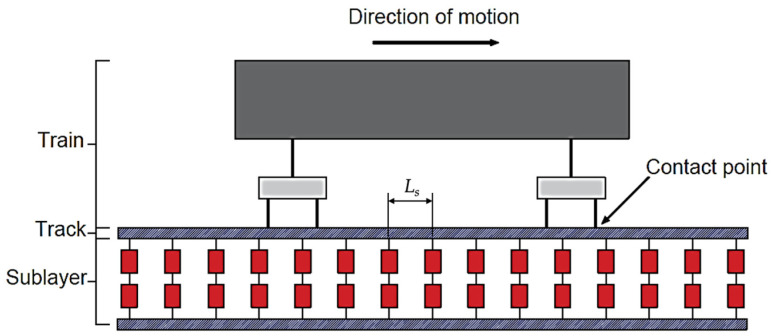
Schematic of the coupled system.

In this paper, a coupled Train–Track Interaction (TTI) model developed by Cantero et al. [45,46] is employed. The TTI model consists of two sub-models—the train and the track (see Figure 1)—which interact and couple at the contact points. These sub-models and the coupling process are explained and illustrated in the following subsections.

### 2.1. Track model

**Figure 2:**
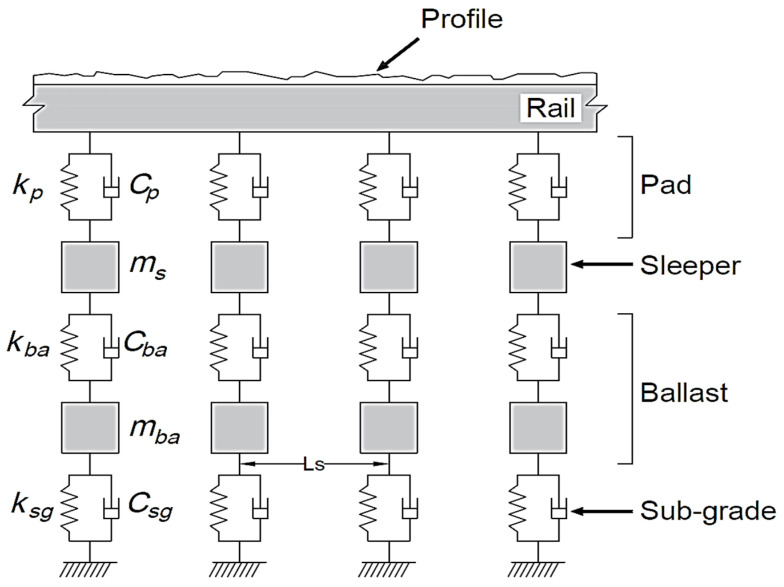
Track numerical model.

Train tracks generally have five components—rails, pads, sleepers, ballast, and subgrade—that can be represented with various levels of sprung layers [45]. In this paper, the track model (see Figure 2) consists of a beam at the surface and three sub-levels modeled by a sprung mass. The beam represents the rail, and the sub-levels represent the sleepers, the ballast, and the subgrade [3,28,47,48,49,50]. The rail beam is created as an Euler–Bernoulli Finite Element (FE) beam, each of which has four degrees of freedom: two translations and two rotations. Two beam elements are modeled between each pair of sleepers with constant properties such as mass per unit length (*m*), modulus of elasticity (*E*), and the second moment of area (*J*). The springs and masses, representing the sub-levels, are located under each sleeper joint spaced at regular intervals of *L_s_*. Geometric and mechanical properties of the track are adapted from Zhai et al. [50] and presented in Table 1.

**Figure 3:**
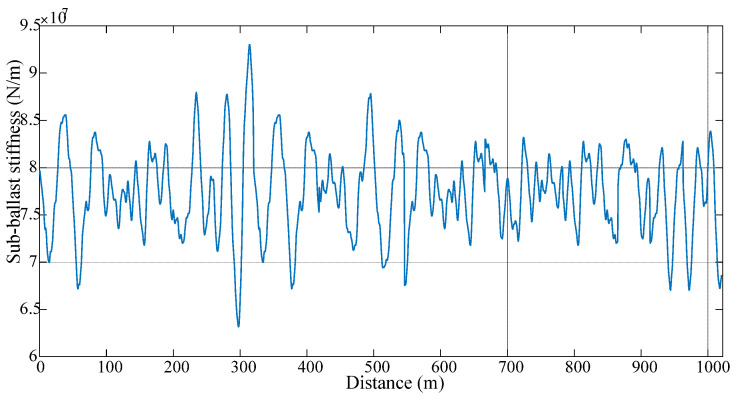
Distribution of the sub-ballast stiffness over distance.

A total length of 1000 m is used for the track, which appropriately allows for continuous welded rails (commonly more than 180 m long [3]). In this paper, a long length of track is considered to maintain stable distance between each end of the boundary conditions. To add realistic parameters to the model, the stiffness of the ballast is considered in a non-uniform way with an average of 7.7495 × 10^7^ N/m and a standard deviation of 4.5824 × 10^6^ N/m. The ballast stiffness, in the space domain, is shown in Figure 3. In addition, a rail profile of class 4 irregularities is generated randomly, according to the US Federal Rail Administration guide. This profile is assumed to be constant in all the simulations in this study. However, in some cases, the profile might change over time, which might not be because of a defect on the track. Therefore, the authors acknowledge that this assumption might not truly reflect real life applications, but it is considered here for simplification. This represents a condition of the surface of the conventional rail, which comes from its power spectral density function [51,52].

The dynamic responses at any location change with time depending on the train position. The FE modeling vectors containing the location of each DOF and its interaction with the corresponding DOFs are created in one matrix, each for stiffness, mass, and damping parameters, using MATLAB software R2021b [53]. Dynamic responses of the modeled track to a time varying force are given by the system of equations at each time-step:

(1) $$M_{t}{\ddot{y}}_{t}+C_{t}{\dot{y}}_{t}+K_{t}y_{t}=f_{int}$$

where $M_{t},$ $C_{t}$, and $K_{t}$ are the mass, damping, and stiffness matrices of the model, respectively; and ${\ddot{y}}_{t},\;{\dot{y}}_{t}$, and $y_{t}$ are the respective vectors of acceleration, velocity, and displacement. $f_{int}$ represents the time-dependent dynamic interaction forces between the train and the track.

### 2.2. Train Model

**Figure 4:**
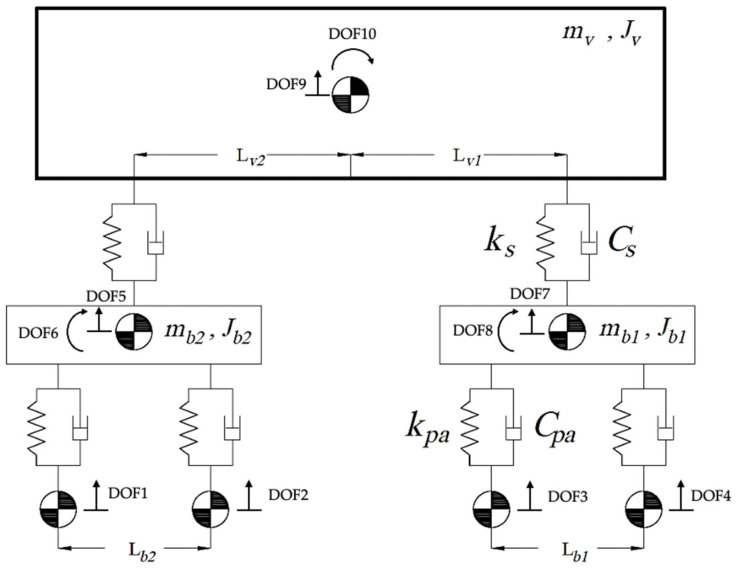
Train half-car numerical model.

A half-train system, as shown in Figure 4, is modeled using MATLAB software R2021b [53] to represent the train. This type of system is adapted from the literature [45,47,50,54], which has been used in multiple TTI-related studies. The train car model has 10 degrees of freedom: 7 vertical translations (four for the wheels, two for the bogies, and one for the main body) and 3 rotations in the plane (two for the bogies and one for the main body). *m_w_*, *m_b_*, and *J_b_* are the mass of the wheelsets, bogie mass, and moment of inertia, respectively; and *m_v_* and *J_v_* represent body mass and its moment of inertia, respectively. Viscous dampers with *c_pa_* damping and a spring with *k_pa_* stiffness are used to connect the wheels with the bogies to form a primary suspension. Likewise, a viscous damper with *C_s_* damping and a spring with *k_s_* stiffness are used for connecting the bogie with the main body. Table 2 shows the mechanical properties of the half-train system, which are adapted from Cantero et al. [45]. It should be noted that in this study, the wheelsets are considered as lumped masses and are assumed to be fixed to the track with no separation being allowed. This means that the system will be modeled as 6 degrees of freedom in the global equations of motion.

The dynamic responses of the vehicle can be measured using the equations of motion represented by the second-order differential equation:

(2) $$M_{v}{\ddot{y}}_{v}+C_{v}{\dot{y}}_{v}+K_{v}y_{v}=f_{v}$$

where $M_{v},$ $C_{v}$, and $K_{v}$ are the mass, damping, and stiffness matrices of the train model, respectively; and ${\ddot{y}}_{v},\;{\dot{y}}_{v}$, and $y_{v}$ represent vectors of train acceleration, velocity, and displacement, respectively. The dynamic interaction forces applied to the vehicle DOFs through the track profile and rail displacements are contained in the vector *f_v_*.

### 2.3. Coupling of Train–Track Models

The train and the track models are combined at the wheels, which represent contact points, to form a coupled TTI system. Equations (1) and (2) are combined in a way that the corresponding DOFs from each model couple together, resulting in global matrices of the system:

(3) $$\left[\begin{array}{cc} M_{v} & 0\\ 0 & M_{t} \end{array}\right]\left[\begin{array}{c} {\ddot{y}}_{v}\\ {\ddot{y}}_{t} \end{array}\right]+\left[\begin{array}{cc} C_{v} & C_{v,t}\\ C_{t,v} & C_{t} \end{array}\right]\left[\begin{array}{c} {\dot{y}}_{v}\\ {\dot{y}}_{t} \end{array}\right]+\left[\begin{array}{cc} K_{v} & K_{v,t}\\ K_{t,v} & K_{t} \end{array}\right][\begin{array}{c} y_{v}\\ y_{t} \end{array}]=F$$

where *M*, *C*, and *K* are global mass, damping, and stiffness matrices, respectively; and *F* is the time-varying vector of interactive force applied by the train to the track. These matrices are calculated at each time-step according to the changing location of the traversing train. Static forces, caused by gravity and the track profile, are also included in the force vector. Equation (3) is solved using the numerical Wilson-Theta integration technique [29]. A value of Wilson-Theta (*θ*) of 1.420815 is used to ensure unconditional stability in the integration process.

### 2.4. Modeling of Damage

In this paper, the percentage loss of track ballast stiffness is used as the damaged condition. A traversing train transfers the moving force through the tracks to the ground, and a change in the stiffness in any component of the system impacts the vehicle’s accelerations [45,47]. Track variability may create a local stress concentration under the sleepers. This could lead to a loss of stiffness under the sleeper and a soft soil formation in the subgrade layer. In the long-term, it may create a loss of contact between the ballast and the sleepers, which are also known as hanging sleepers [55]. If hanging sleepers are not detected at the right time, they may accelerate the track deterioration and cause more damage. In the numerical model, the damage is simulated by reducing the ballast stiffness by 10%, 30%, 50%, and 70% of the healthy state. These damage conditions represent partial or nearly complete loss of the ballast underneath the track, which is one of the major issues commonly seen by the track inspectors [3,24]. The acceleration response from the traversing train is measured using health and damaged track cases and compared using the machine learning and data-driven approach.

## 3. The Proposed Algorithm for Track Monitoring

**Figure 5:**
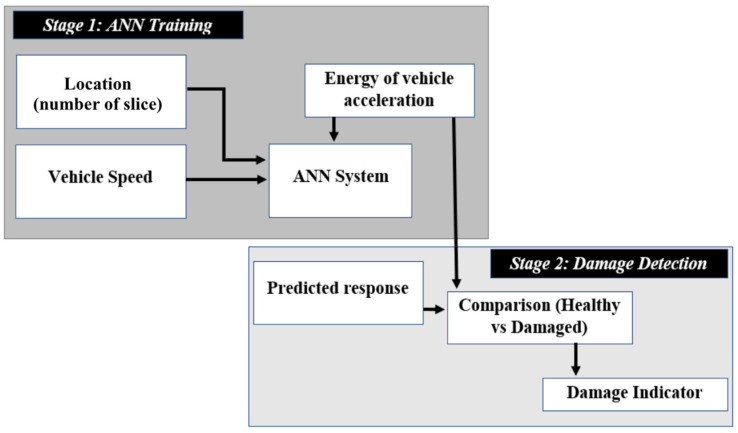
The proposed machine learning approach.

A novel track monitoring algorithm is proposed in this paper that consists of two phases in correlation to detect track damages. The first phase utilizes an ANN model to estimate the energy of train acceleration responses while transversing the irregular track with healthy conditions. This involves training a neural network to predict data from simulated acceleration measurements. In the second phase, a test data point is predicted using the neural network and compared with the simulated data. Also, the healthy and the damaged data cases are separated and compared to show the changes in the predicted data from the trained ANN. These two phases are illustrated as a flowchart in Figure 5, which shows signal processing steps involved in each phase.

The ANN is a commonly used algorithm for monitoring structures and is used to predict the energy content of an in-service train response, considering various energy bands and train speeds. During the monitoring stage (phase 2), the calculated energy spectrum from each vehicle passage is compared to that predicted by the ANN and a damage indicator (DI) is evaluated for each traversing train.

### 3.1. ANN Background

A neural network is a very useful and evolving tool for predicting one or more desired outcomes in complex systems, using a history of data collection and previous outcomes. This tool has been used several times in research studies to solve predictions to nonlinear problems, pattern recognition, or optimization [30,39,56]. Neural networks consist of incoming data, hidden data, outgoing data, weights and bias, an activation function, and a summing node [36,57]. Each level incorporates several units of calculation called a neuron [30,41], which takes its input data from the previous level and provides output data for the next level. The input level provides the input data of the network, which are fed to the hidden level. Each hidden level has a number of neurons that will calculate an output using all the inputs of the input level and a predefined set of weights and bias. This output can be either fed to the next level or directly to the output layer. The output level analyzes all the input produced in the last hidden level and produces the last output of the whole algorithm.

**Figure 6:**
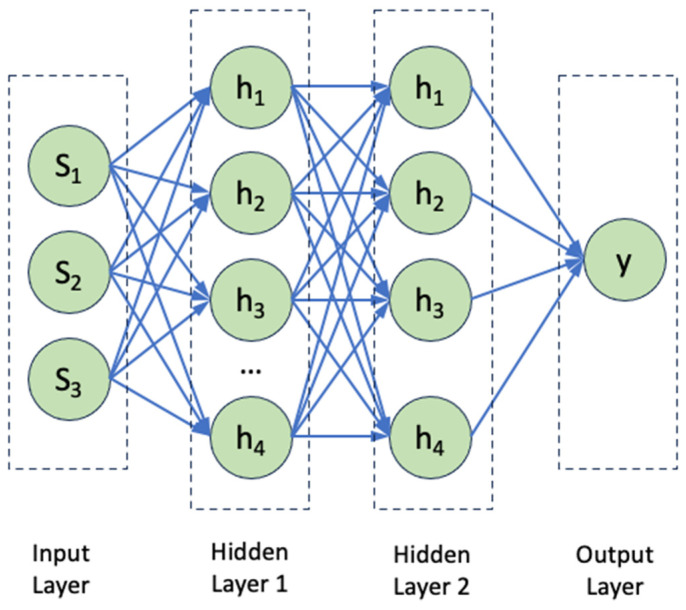
Flowchart of output calculations using neurons.

Using the MATLAB deep learning toolbox, the ANN is implemented in this paper to a set of vehicle acceleration data. The ANN consists of an input layer with 3 input neurons, two hidden layers (containing 20 neurons each), and an output layer. The output layer provides the predicted results that, in this case, is the predicted energy response. Figure 6 shows the schematic of the ANN where each neuron calculates a single output data point from the inputs at the previous levels. In Figure 6, *S_i_* is the output from the neuron *n_i_* of the previous level, *w_i_* is the weight associated with *n_i_*, *b* is the neuron bias, and *S* is some transform function, typically sigmoid. Activation and transform functions are given in Equations (4) and (5), respectively. The main role of the activation function is to transform the summed weighted input into an output value, which will be used to feed into the next hidden layer.

(4) $$\mathrm{Activation}:\;\sum_{i=1}^{N}w_{i}S_{i}$$

(5) $$\mathrm{Transform}:\;S(b+\sum_{i=1}^{N}w_{i}S_{i})$$

In this paper, the ANN is implemented using the supervised learning approach, which is based on the difference between the predicted and the simulated outputs. To train the ANN model, a Levenberg–Marquardt backpropagation (LMBP) algorithm [58] is applied, which operates in a closed loop to minimize the differences between the predicted and measured signals. The hidden layers of the ANN contain two hyperbolic tangent (TanH) activation functions, and the output layer contains a linear activation function. In this process, the ANN must reach a state of τ* = τ, where τ* is the vector of optimized parameters and *τ* is the vector of neural network parameters. The number of parameters depends on the number of neurons, at each sub-level of learning. As described in Equation (6), for a couple (*s*, *y*), the *arg* operator minimizes the value predicted by the neural network using the input data and the actually expected data to calculate the τ* value:

(6) $$\;\;\;\;\tau^{*}=arg\left\{\left|\mathrm{ANN}\left(\tau ,s_{i}\right)-y_{i}\right|\right\}$$

Hidden layers perform as neuron nodes in-between inputs and outputs, which allow the neural network to learn complicated features. This is performed by the multiple neurons in each hidden layer that carry an assigned function and weight, depending on the errors coming from each iteration. The correct number of hidden levels and neurons is necessary to achieve accurate results with the least computational effort required. The LMBP process is applied to the ANN, in which, during training, random weights are assigned to the neurons initially, the inputs are passed through the hidden level to give a predicted output value, and an error value is calculated between the predicted and actual output value. An iterative process is created that adjusts the weight at each cycle to minimize the error. LMBP facilitates convergence to a stable system with low computational effort and time, and it combines the aspects of the steepest-descent method and the Gauss–Newton method to improve the accuracy of the output with fewer iterations required [58,59]. Therefore, the LMBP algorithm is used in this paper to utilize the ANN architecture more efficiently.

### 3.2. The Proposed ANN Model

A novel ANN model is developed that predicts the energy level of an in-service train acceleration, given that the speed of the train is known. For the train response, the energy of the vertical acceleration signal of the first bogie is chosen, as it weighs uniformly and consistently for most of the trains due to engine weight. The acceleration signals are transformed from the time domain to the space domain using the location and speed of the train. The space domain is then divided into several segments of the same size where the energy of the signals is calculated for each segment. The number of segments is used as the second input in the ANN. The energy of the vertical acceleration signal will be summed up for each segment and will used as the output.

(7) $$\;e_{k,j}={\ddot{u}}_{k,j}{}^{2}$$

where $e_{k,j}$ is the energy of spatial point *k* for run *j* and ${\ddot{u}}_{k,j}$ is the vertical acceleration of spatial point *k* for run *j*.

The independent variable *s* is formed of the vector containing

(8) $$s_{i,j}=\left(i,v_{j}\right)$$

where *i* is the segment number (so the spatial location) and *v* is the speed, constant over the whole passage, for run *j*.

The dependent variable can be translated to

(9) $$E_{i,j}=\sum_{I}e_{k,j}$$

where *I* represents the set of points included in segment *i, e* is the energy at special point *k*, and *j* represents the number of the run.

### 3.3. Damage Indicator

The damage indicator is formed using the prediction error, which is the square root of the difference between the predicted and simulated data for each segment and each pass.

(10) $$Pe_{i,j}={\left\{\mathrm{ANN}\left(\tau^{*},s_{i,j}\right)-E_{i,j}\right\}}^{2}$$

where *Pe* is the prediction error of segment *i* and run *j*, and ANN is the operator that predicts the data using the ANN trained in the training phase.

The prediction error might vary significantly depending on the speed of the train passage. This results from a stochastic distribution that is characterized by a normal distribution to recognize the healthy structure from the damaged one. In this article, prediction errors are considered to follow a Gaussian process with a mean of µ and standard deviation of σ.

(11) $$Pe_{i,j}\sim \left(µ,\sigma \right)$$

The prediction errors for a healthy structure will be quite low, and they will remain low as long as a healthy structure is predicted. This means that the energy predicted by the ANN is at the same level as the energy measured by the train during the testing phase. A damage indicator is then introduced by comparing the prediction error with the mean of the prediction errors of the healthy structure, divided by the standard deviation of the prediction errors of the healthy structure.

(12) $$DI_{i,j}=\frac{Pe_{i,j}-{µ}_{training}}{\sigma_{training}}$$

where ${µ}_{training}$ and $\sigma_{training}$ are, respectively, the mean and the standard deviation of the prediction errors of the healthy structure.

## 4. Result of the Machine Learning

**Figure 7:**
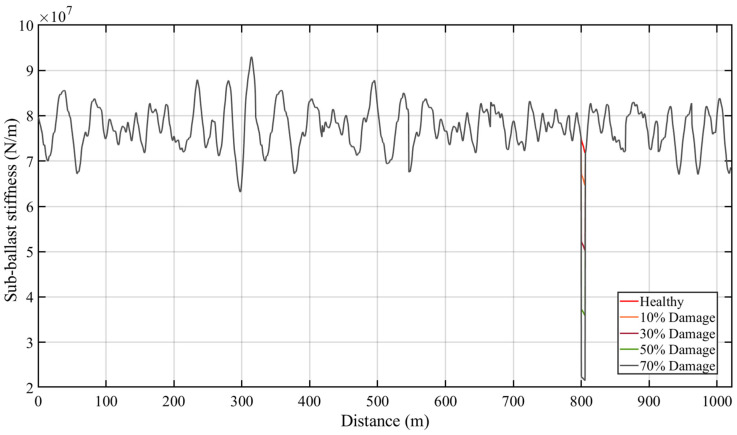
Sub-ballast stiffness profile with different damaged conditions at 800–805 m section.

In this section, the proposed ANN model is analyzed using the numerical TTI model shown in Section 2. A 1100 m long track is modeled and a track section in between 700 m and 1000 m is used to test the proposed algorithm to avoid the boundary conditions and to stabilize the DOFs. The damage is modeled as a loss of stiffness of the ballast, and four damaged cases are simulated. These include reductions in stiffness down to 10%, 30%, 50%, and 70% of the healthy stiffness. The damage is simulated as a local loss and a 5 m section (from 800 m to 805 m length) is chosen to assess the efficacy of the approach. Figure 7 illustrates the stiffness of the ballast with healthy and damaged case scenarios.

**Figure 8:**
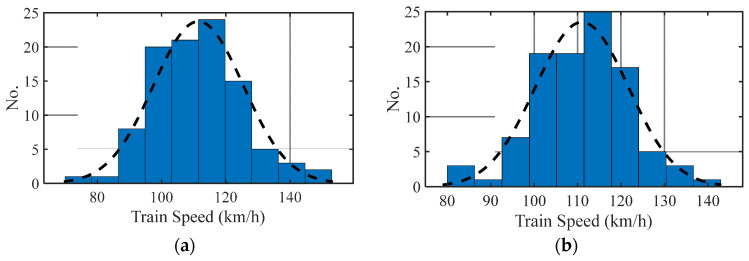
Distribution of train velocities for (**a**) healthy case and (**b**) damaged case.

A fleet of trains traversing the track with the roughness profile is simulated, and the train bogie acceleration energies are calculated using a sampling rate of 500 Hz. For a healthy case, a fleet of 100 trains is selected, which is used to train the proposed ANN model. For each run, the train velocity is chosen randomly following a normal distribution with a mean of 110 km/h and a standard deviation of 12 km/h. Similarly, a fleet of the same size each is used for the four damaged cases. The distributions of randomly chosen velocities for the healthy and damaged cases are shown in Figure 8a,b. In this section, no signal noise is added in the simulations and the energy magnitudes are calculated for each time step.

The healthy track case is used to train the ANN model, which adjusts and stabilizes the weights of the neurons in each layer. The trained ANN model is used to predict the output data for the next four fleets of the damaged cases. These predicted data are compared with the simulated data and the error and damage indicators are calculated. In this section, 300 m of the track around the damage location is chosen and sliced into segments of 15 m (resulting in 20 slices), and the energy of the vertical acceleration of the train is predicted and compared with the interval of each segment.

**Figure 9:**
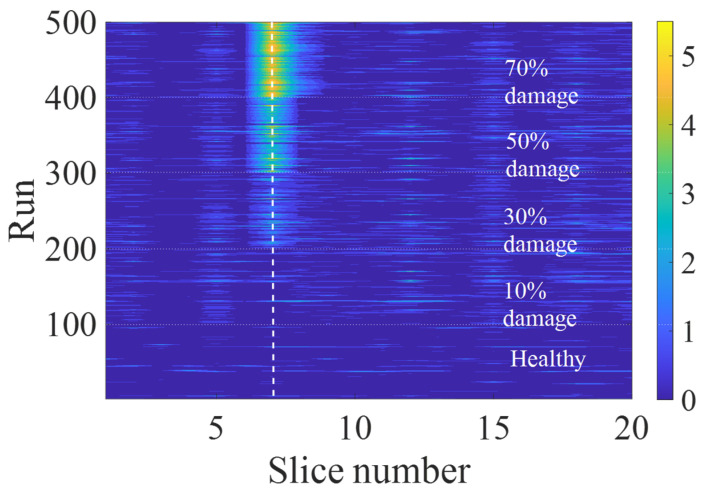
Damaged indicator by slice and run-on logarithm scale (contour plot).

The change in the damage indicator with the change in damage percentage is illustrated as a contour plot in Figure 9 on a logarithmic scale that allows us to make a better observation of the energy behavior with damage. In this figure, the number of runs is indicative of one healthy and four damaged cases, meaning that the first 100 runs represent the healthy condition, 101–200 runs represent the 10% damaged condition, 201–300 runs represent the 30% damaged condition, 301–400 runs represent the 50% condition, and 401–500 runs correspond to the 70% damaged condition. The runs are not sorted according to the velocities and are arranged randomly as chosen. It is also noted that a lower damage percentage, e.g., 10% loss of stiffness, is difficult to detect using the simple ANN analysis, and more research would be required for detecting a low magnitude of damages. However, the proposed ANN model can be a suitable system to identify the damage before any critical event and help prevent serious consequences.

## 5. Sensitivity Analysis

In this section, the sensitivity analysis is also carried out to ensure robustness of the ANN model. Three new scenarios are simulated, and the analysis is repeated to assess the efficacy of the proposed system: (i) change in energy band, (ii) adding signal noise, and (iii) multiple damage locations. In the previous analysis, the healthy and damaged tracks were sliced with a size of 15 m.

### 5.1. Size of Segments

**Figure 10:**
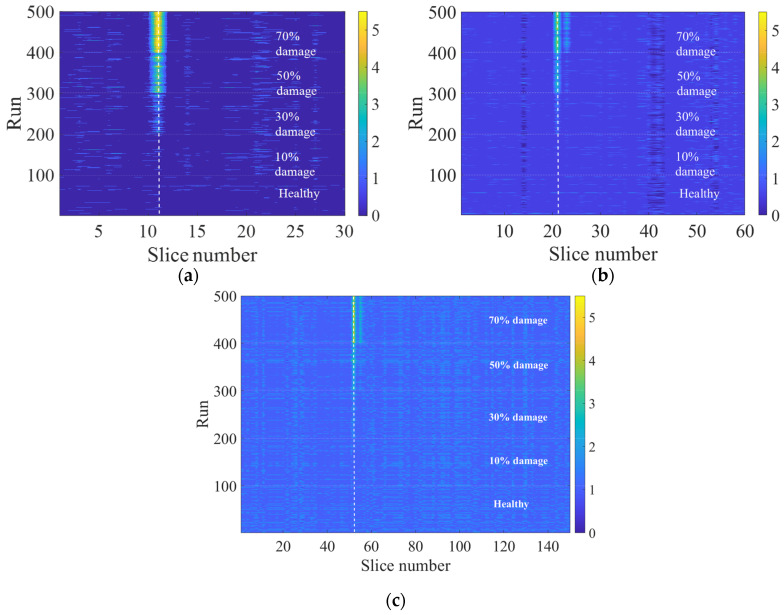
Damaged indicator by slice and run-on logarithm scale (contour plot): (**a**) 10 m, (**b**) 5 m, (**c**) 2 m.

In this analysis, the impact of the size of the slices on the effectiveness of the proposed approach is studied. The analysis in the previous section is repeated with three different lengths of segments—(i) 30 slices of 10 m, (ii) 60 slices of 5 m, and (iii) 150 slices of 2 m. The damage location and the train properties are kept similar to the previous analysis, and the performance of the proposed model is assessed using finer sizes of segments. This is important to see the ability of the ANN model to identify the location of the damage and its magnitude with a changing level of computational effort. Figure 10a–c illustrate the results of the first sensitivity analysis where three different lengths of segments are used. It can be seen in Figure 10b,c that with a smaller segment size (finer segment), there is a loss of precision and clarity in the results. It should be noted that there is expected to be a trade-off between the size of the area being affected on the track and the size of slices. In this case, the damaged segment is on a 5 m section of the track; however, it can be seen that a slice size of 10 m shows better results compared to the 2 m. This can be considered as an important finding when it comes to real-life applications of the method.

This may be caused by the comparison between the damaged area length and the size of the segment, e.g., as shown in Figure 10c where the size of the segments is less than the actual length of the damaged area.

### 5.2. Noise Assessment

A second sensitivity analysis is carried out by repeating the analysis from Section 4 with the addition of random noise in the acceleration signal. In field testing, sensors tend to have a certain magnitude of imperfection, which results in random perturbations in the signals. In addition, other sources of error such as changes in rail roughness profile and the temperature effects can be considered as sources of noise. In order to evaluate the impact of these parameters on the accuracy of the results, the noisy signal is generated using a commonly used equation (Equation (13)) that creates a white noise vector randomly using a normal distribution with a mean of 0 and a standard deviation of a percentage of the signal amplitude [60,61]. The white noise vector is then added to the calculated responses to generate noisy/polluted responses. It can be mathematically represented as

(13) $${\ddot{u}}^{polluted}=\ddot{u}+E_{p}\times N_{noise}\times \sigma \left(\ddot{u}\right)$$

where $\ddot{u}$ is the calculated response and $\sigma \left(\ddot{u}\right)$ is its standard deviation, $N_{noise}$ is a standard normal distribution vector with zero mean value and unit standard deviation, $E_{p}$ is the noise level, and ${\ddot{u}}^{polluted}$ is the polluted response of the model. For the analysis in this section, two percentages of the standard deviation 5% and 10% are used to add the noise to the train bogie accelerations.

**Figure 11:**
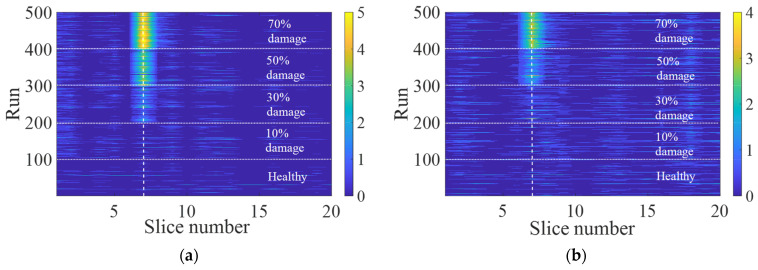
Influence of the noise on the DI (plotted on logarithmic scale): (**a**) with 5% signal noise and (**b**) with 10% signal noise.

For each noise level, a new set of healthy track data using noisy accelerations is measured to train the ANN model. Also, a set of 400 runs are simulated for the damaged cases to assess the damage indicator with added signal noise. The contour plots of the damage indicators from the analysis, using the noisy signals, are shown in Figure 11, where Figure 11a,b represent the results with 5% and 10% noise in the signals, respectively. It can be seen from Figure 11 that the proposed ANN model for monitoring the sub-ballast stiffness of the tracks works with reasonable results and is reliable under realistic conditions.

### 5.3. Multiple Damage Locations

**Figure 12:**
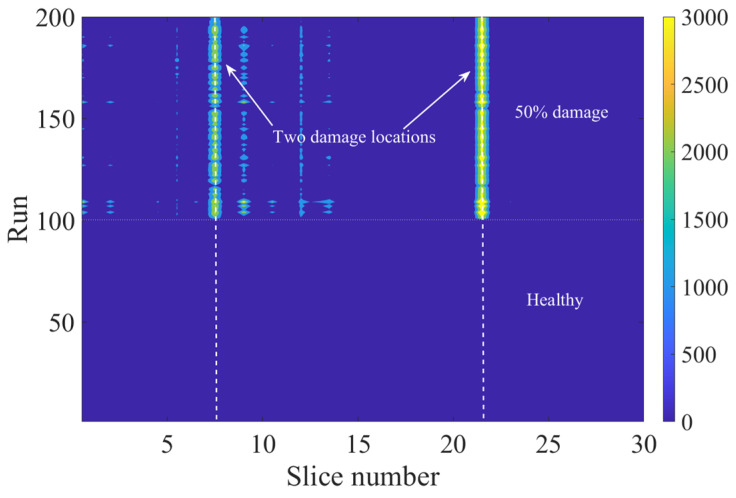
The results of the damage indicator with two damaged sections: 800–805 m (slice 8) and 950–955 m (slice 22).

In this section, the proposed approach is also tested with damages at multiple locations. In this analysis, two sections of the track are considered with a 50% loss of ballast stiffness. The sections are 800 m to 805 m and 950 m to 955 mm, and a slice length of 5 m is chosen for the analysis. Figure 12 illustrates the results of the proposed ANN analysis damage indicator with two damaged locations. It can be seen in the figure that there is a significant increase in the signal energy differences at the two damaged locations (slice 20 and 50, respectively). Although there are some other visible slices that have shown a change in magnitude of damage indicator, these differences are not as significant as the differences at the damaged slices. This analysis proves that the proposed approach is effective even if there are multiple locations of damage.

## 6. Conclusions

A novel railway track damage detection approach is proposed in this paper using a machine learning technique that combines an Artificial Neural Network model (ANN) and a Gaussian process to detect the loss of track sub-ballast stiffness. The ANN is trained using energy responses of 100 simulated vertical train accelerations traversing over a healthy track. Using the trained ANN, the energy responses are predicted and the prediction error for each passage of trains is calculated using the square of the difference between the simulated and the predicted responses. The prediction error is assessed using different track sub-ballast stiffnesses and a Damage Indicator (DI) based on the prediction error is proposed. In order to interpret the prediction errors and to minimize the error in the machine learning process, the DI is defined using a Gaussian process and is used to normalize the distribution of the prediction errors. The numerical study demonstrates that this novel approach is effective in detecting changes in sub-ballast stiffness and is able to locate the area of damage. Although the approach is tested for the sub-ballast stiffness loss, other types of rail damages may also be monitored (by training the algorithm with different damage cases) and therefore will be part of our future studies. This paper provides a theoretical concept and numerical validation for track damage detection using the ANN. However, a full-scale real-life demonstration of the approach is recommended as part of future work to test the resilience of the approach on real-life tracks where environmental variations and other physical phenomena might limit the effectiveness. A high-accuracy positioning system, to record the train location in time and to calculate the average speed, is an essential element in such installations. In addition, the rail and track profile are assumed to be constant during the training and testing phase. However, it should be noted this will not be necessary in real-life applications. Therefore, further studies need to be carried out to address this drawback.

## Figures and Tables

**Table 1:** Properties of the track.

Property	Unit	Value
Elastic modulus of rail	N/m^2^	2.059 × 10^11^
Rail cross-sectional area	m^2^	1
Rail second moment of area	m^4^	3.217 × 10^−5^
Rail mass per unit length	kg/m	60.64
Rail pad stiffness	N/m	6.5 × 10^7^
Rail pad damping	Ns/m	7.5 × 10^4^
Sleeper mass (half)	kg	125.5
Sleeper spacing	m	0.545
Ballast stiffness	N/m	137.75 × 10^6^
Ballast damping	Ns/m	5.88 × 10^4^
Ballast mass	kg	531.4
Subgrade stiffness mean	N/m	77.5 × 10^6^
Subgrade damping	Ns/m	3.115 × 10^4^

**Table 2:** Properties of the train.

Property	Symbol	Unit	Value
Wheelset mass	*m_w_*	kg	1843.5
Bogie mass	*m_b_*	kg	59,364.2
Car body mass	*m_v_*	kg	5630.8
Moment of inertia of bogie	*J_b_*	kg·m^2^	9487
Moment of inertia of main body	*J_v_*	kg·m^2^	1.723 × 10^6^
Primary suspension stiffness	*k_pa_*	N/m	2.399 × 10^6^
Secondary suspension stiffness	*k_s_*	N/m	0.8858 × 10^6^
Primary suspension damping	*c_pa_*	Ns/m	30 × 10^3^
Secondary suspension damping	*C_s_*	Ns/m	45 × 10^3^
Distance between car body center of mass and bogie pivot	*L_v_*_1_, *L_v_*_2_	m	5.73
Distance between axles	*L_b_*_1_, *L_b_*_2_	m	3

## Data Availability

Not applicable.
